# Supplementary material for: EGFR Transgene Stimulates Spontaneous Formation of MCF7 Breast Cancer Cells Spheroids with Partly Loss of HER3 Receptor
Source: Int J Mol Sci. 2021 Nov 29;22(23):12937. doi: 10.3390/ijms222312937 (PMC8657849; doi:10.3390/ijms222312937)

Figure S1. Sensitivity of MCF7wt and MCF7-EGFR cells to EGFR inhibitor AG1478. Data of MTT test, 72 h after the treatment.

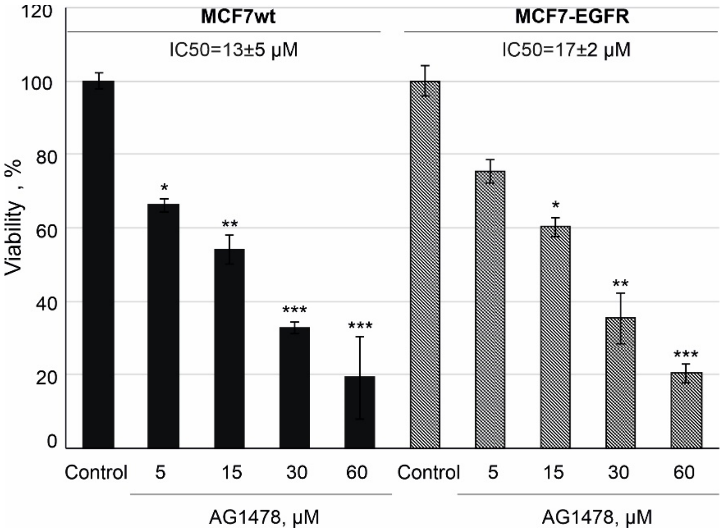

Figure S2. HEK 293T- EGFR cells. (A) Typical picture of HEK 293T and HEK 293T- EGFR cells. (B) Analysis of CD44 and CD24 receptors in HEK 293T and HEK 293T- EGFR cells.

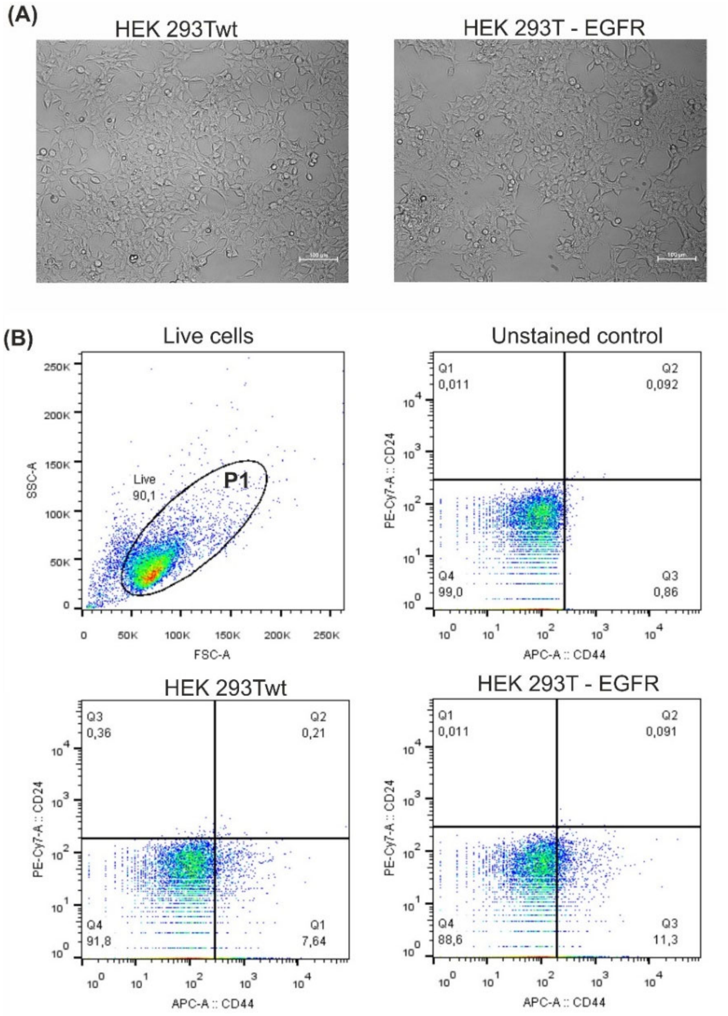

Figure S3. Typical analysis of HER3 receptors by flow cytometry. Q1 – HER3-positive cells, HER3^{bright} cells are marked by a blue rectangle.

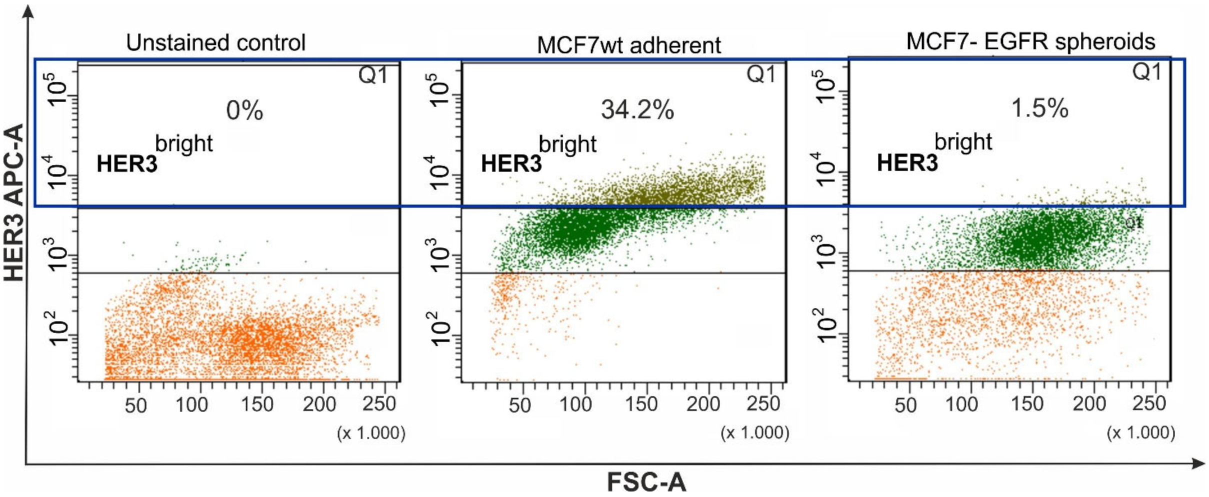

Supplement: Supplementary file 1 [file ijms-22-12937-s001.zip › ijms-1437297-supplementary.pdf]
